# African vaccine manufacturing – The ecosystem and the Initiative

**DOI:** 10.4102/jphia.v15i1.642

**Published:** 2024-08-22

**Authors:** Tolu Disu, Charles Kamau, Lisa Bonadonna, Farrah Losper, William Ampofo

**Affiliations:** 1 African Vaccine Manufacturing Initiative (AVMI), London, United Kingdom; 2 African Vaccine Manufacturing Initiative (AVMI), Nairobi, Kenya; 3 African Vaccine Manufacturing Initiative (AVMI), Cape Town, South Africa; 4 African Vaccine Manufacturing Initiative (AVMI), Accra, Ghana

Dear Editor,

Africa stands at a pivotal juncture in its vaccine manufacturing journey. With a growing population, a persistent burden of vaccine-preventable diseases, and an overdependence on foreign shots, the imperative to secure health equitably through the manufacture of vaccines is now a social, political, and economic imperative for the continent as well as a pandemic preparedness necessity for Africa and the world.^1,2^ Since 2010, the African Vaccine Manufacturing Initiative (AVMI) has championed the cause for local vaccine production, supported by in-Africa research and development (R&D). From ecosystem collaborator and partnership facilitator to industry organisation and strategy contributor,^3^ AVMI continues to advocate for and contribute to (unpublished) the African Union vision for the African vaccine manufacturing industry to develop, produce, and supply over 60% of the total vaccine doses required on the continent by 2040.^4^ African Vaccine Manufacturing Initiative is striving to achieve this with a vibrant and sustainable high-value manufacturing and biomedical ecosystem involving all the regions of Africa.

## African vaccine ecosystem platform

The African continent has a critical need for a robust and collaborative ecosystem given the plethora of projects announced in countries across Africa. Stakeholders, including funders, technology contributors and market access facilitators, must work together if the vision of African-vaccines-for-Africa is to be realised.^5,6,7,8^ A convergence within the ecosystem is crucial to:

- overcome fragmentation which hinders knowledge sharing, collaboration, and resource optimisation^2,3^
- unite diverse groups to amplify the collective voice that strengthens advocacy efforts for fairer access to technology, funding, and policy changes for all
- showcase Africa’s collective potential to de-risk investments and unlock critical funding across the vaccine value chain
- focus on building a sustainable economic future that fosters long-term planning and coordinated action.

Nestled within this ecosystem is AVMI’s intimacy with the African context and rapidly evolving local environment as well as the organisation’s proven ability to work with supranational and international stakeholders key to the design, implementation, funding, and sustainability of vaccine manufacturing. Accordingly, AVMI aligns and supports the aims of the Africa Centres for Disease Control and Prevention (Africa CDC) Partnership for African Vaccine Manufacturing (PAVM) and industry. Between African manufacturers and policy makers, AVMI fosters a conducive environment for vaccine production through dialogue and knowledge exchange leading to informed frameworks and policies. Among researchers and developers and regulatory authorities, AVMI’s thought leadership and engagement seek to expedite approvals and clinical development harmonisation. And on the political front, AVMI advocates for African investment from its own members and a commitment to ‘buy African’ for a long-lasting sector. A case in point for the showcasing of these competencies took place at our previous Sustainable Investments for African Vaccine Manufacturing meeting and workshop in Addis Ababa (unpublished).

## Voice of African vaccine industry

On the global stage, the African industry is gaining momentum especially post-coronavirus disease 2019 (COVID-19), but fragmentation could threaten progress, given some 30 distinct vaccine projects have already been announced.^2,3^ While individual manufacturers are making strides, a unified voice is critical in overcoming challenges and securing a sustainable future, the latter being a crucial consideration not just for producers but for organisations such as the Global Alliance for Vaccines and Immunisation (GAVI), the Africa CDC, and others.

African Vaccine Manufacturing Initiative currently has over 25 prospective and current manufacturer members spread across Africa and represents the interests and perspectives of its diverse constituents. Addressing industry pain points relating to technology transfer, regulatory requirements, and market demand shaping, the initiative provided defining input into GAVI’s African Vaccine Manufacturing Accelerator (AVMA).

An innovative financing instrument developed in close consultation with the Africa CDC, the AVMA aims to support a sustainable and resilient African vaccine manufacturing industry. With $1 billion committed to aiding producers across the development and manufacturing spectrum, it is a concrete move to bolster regional health emergencies preparedness and enhance global vaccine markets. Industry can potentially access the facility via two modes of AVMA payments: World Health Organization Pre-Qualification (WHO PQ) milestones and successful United Nations International Children’s Emergency Fund (UNICEF) tender arrangements.^9,10^

Beyond direct benefits to individual local vaccine players, this unique facility is prospectively positive for the entire ecosystem; African countries and constituents would eventually gain by having more readily available and affordable vaccines adapted for endemic and emergency scenarios against a backdrop of job creation and economic growth.

Indeed, the development is ground-breaking, though fine-tuning of the AVMA instrument would allow for better industry–market harmonisation. By recommending a focus on scope (broadening the range of covered vaccines), scale (increasing the fill and finish component in the short-term), and advocacy (ensuring the WHO PQ process is supportive of the instrument), AVMI voices additional specific industry needs of the AVMA (unpublished). Table 1 presents these recommendations and related justifications. Collectively, such measures would allow for cost-competitiveness, incentivised technology transfers, creation of viable markets, and a return on investment to GAVI funders and investors.

**TABLE 1:** Themes, associated recommendations and rationales for Global Alliance for Vaccines and Immunisation African Vaccine Manufacturing Accelerator from African Vaccine Manufacturing Initiative, the voice of industry.

Theme	Recommendation	Rationale
Scope	Broaden the range of vaccines covered by the AVMA	Mitigates risk of over investment in designated GAVI priority antigens Promotes more equal opportunity for tech transfer and tendering for vaccine development Allows African manufacturers to participate in existing ‘higher volume’ segments thereby contributing to globally healthier markets, technological access and outbreak preparedness
Scale	Increase the fill and finish component in the short-term of AVMA	Serves as a stop-gap measure for vaccine market sustainability, in the absence of demand allocation (volumes) and demand certainty (long-term and/or offtake agreements) Provides an incentive and/or subsidy for the regional industry to be more cost-competitive, through economies of scale, at the end of the AVMA period Utilises funding within a 10-year period as market works towards longer term drug substance capacity and capability-building
Advocacy	Ensure the WHO PQ process supports the AVMA	Complements GAVI’s traditional roles of collaborating with multilateral and domestic partners for operational industry support and national-led product demand, respectively Promotes alignment of regulatory and procurement cycles for impact effectiveness of the instrument

AVMA, African Vaccine Manufacturing Accelerator; GAVI, Global Alliance for Vaccines and Immunisation; WHO PQ, World Health Organization Pre-qualification.

Furthermore, the AVMI position paper on the Prioritisation of Regulatory Enablers posits for the reinforcing of national and regional regulatory agencies through attainment of WHO Maturity Level 3 status, capability-building of the Regional Centres of Regulatory Excellence (RCOREs), and operationalisation of the African Medicines Agency (AMA) and the African Medicines Regulatory Harmonisation Programme (AMRH), all aligned with the Africa CDC PAVM Framework for Action (FFA). The enhancement of WHO PQ procedure to quicken the inclusion of quality Africa-made vaccines is also cited to promote the global regulatory environment (unpublished). In summary, regulatory strengthening at all levels within the African vaccine ecosphere is critical to the development, production, and supply of high-calibre and safe medicines and their associated acceptance worldwide.

## African Vaccine Manufacturing Initiative and Africa Centres for Disease Control and Prevention Partnership for African Vaccine Manufacturing: A symphony of strengths

The vision for a durable and localised African vaccine value chain cannot be fully realised without coordination and strategy underpinned by unwavering political will. This is where Africa CDC and AVMI are well-placed to provide broad and representative leadership for actors and actions in regional ecosystems, while supporting individual manufacturers to chart their own courses. In addition to bridging information gaps and providing strategic direction,^4^ the continental coordinator role mitigates the risks of specific overcapacity, general oversupply, and cannibalisation of vaccine products. During the early days of PAVM’s formation, AVMI played a role as a consultant, contributor, and champion for its establishment. The intertwined roles and paths of AVMI and Africa CDC’s PAVM are critical in driving the journey towards Africa’s vaccine self-sufficiency and in creating an ecosystem of support and alliance. Africa CDC and AVMI working collaboratively have convening capacity over a wide range of local, regional, continental, and global stakeholders critical to the development of a sustainable African vaccine ecosystem. Together these two entities can underscore and make undeniable key priorities such as harmonising the regulatory landscape for a predictable and high-quality environment, and easing access to markets, through regional economic blocs, to create a synergistic impact and promote vaccine equity. The mutual respect and trust between Africa CDC and AVMI have enabled advancement of the technology transfer and intellectual property unit, major contributions to the R&D, talent development, market design and demand intelligence, and infrastructure progression within the eight bold programmes in PAVM’s FFA.

## African Vaccine Manufacturing Initiative: The source

Coordination and convention provide the platform for knowledge exchange. African Vaccine Manufacturing Initiative is not just gathering information from the ecosystem, it is illuminating the path towards African vaccine self-sufficiency and taking on the crucial role as a credible information pathway notably through its industry forum. Curation of Africa-specific vaccine manufacturing knowledge from conference proceedings, original and secondary thought leadership articles, and production of white papers and relevant workshop reports have proven formidable. Through online platforms such as AVMI-member communications, sole and joint webinars, meetings and guest-speaking opportunities by AVMI Board members, social media and key events, partnerships are fostered, and insights are continuously exchanged.

## Looking ahead

African Vaccine Manufacturing Initiative’s future is about empowering Africa’s vaccine journey. Moving forward, AVMI seeks to remain focused on advocacy, market intelligence and access, partnership fostering, diverse stakeholders’ bridge-building, and industry representation. African Vaccine Manufacturing Initiative strives to stay adaptable and responsive to addressing the changing needs of the continent and global landscape, in relation to safeguarding health equity and security, through African delivered, manufactured, and ultimately developed and researched vaccines.^11^

For the next period, the AVMI priorities include continuing to champion a fit-for-purpose AVMA, advocating for an expedient AMA establishment and a progressive WHO PQ process, and working with Africa CDC, member states, and all key stakeholders on instruments to unlock continental demand including a pooled procurement mechanism. Ultimately, AVMI would be shaped by its core as a catalyst and an enabler in the African vaccine manufacturing ecosystem, and guided by its values of transparency, integrity, collaboration, and trust.

The authors acknowledge the contributions of the AVMI Working Groups and the Executive Board for personal communications/unpublished works. In addition to the stated membership of the AVMI, the authors declare affiliations with the African vaccine manufacturing industry through employment and other capacities/relationships.
